# New Insights into Long Terminal Repeat Retrotransposons in Mulberry Species

**DOI:** 10.3390/genes10040285

**Published:** 2019-04-09

**Authors:** Bi Ma, Lulu Kuang, Youchao Xin, Ningjia He

**Affiliations:** State Key Laboratory of Silkworm Genome Biology, Southwest University, Beibei, Chongqing 400715, China; mbzls@swu.edu.cn (B.M.); miffyjolia@sina.com (L.K.); mulberry201@email.swu.edu.cn (Y.X.)

**Keywords:** Long terminal repeat (LTR) retrotransposons, *Morus notabilis*, insertion time, *Copia*, *Gypsy*, transposable elements

## Abstract

The evolutionary dynamics of long terminal repeat (LTR) retrotransposons in tree genomes has remained largely unknown. The availability of the complete genome sequences of the mulberry tree (*Morus notabilis*) has offered an unprecedented opportunity for us to characterize these retrotransposon elements. We investigated 202 and 114 families of *Copia* and *Gypsy* superfamilies, respectively, comprising 2916 intact elements in the mulberry genome. The tRNA*_Met_* was the most frequently used type of tRNA in both superfamilies. Phylogenetic analysis suggested that *Copia* and *Gypsy* from mulberry can be grouped into eight and six lineages, respectively. All previously characterized families of such elements could also be found in the mulberry genome. About 95% of the identified *Copia* and *Gypsy* full elements were estimated to have been inserted into the mulberry genome within the past 2–3 million years. Meanwhile, the estimated insertion times of members of the three most abundant families of the *Copia* superfamily (908 members from the three most abundant families) and *Gypsy* superfamily (783 members from the three most abundant families) revealed divergent life histories. Compared with the situation in *Gypsy* elements, three families of *Copia* elements are under positive selection pressure, which suggested that *Copia* elements may have a dominant influence in the evolution of mulberry genes. Analysis of insertion and deletion dynamics suggested that *Copia* and *Gypsy* elements exhibited a very long half-life in the mulberry genome. The present work provides new insights into the insertion and deletion dynamics of LTR retrotransposons, and it will greatly improve our understanding of the important roles transposable elements play in the architecture of the mulberry genome.

## 1. Introduction

Transposable elements (TEs) are mobile genomic DNA sequences that have been proven to be ubiquitous and abundant components in almost all eukaryotic genomes so far, and they play important roles in the evolution and structural organization of genes and genomes [1,2,3,4]. Long terminal repeat (LTR) retrotransposons, one of the classes of TEs, are amplified through a “copy and paste” method in their host genome [5]. Typical characteristics of an intact LTR retrotransposon include: (1) two identical LTRs; (2) a PBS site (primer-binding site); (3) a PPT tract (polypurine tract); (4) a *Gag* gene, which encodes a polyprotein; and (5) a *Pol* gene, which encodes several domains, including RT (reverse transcriptase), RH (RNase H), IN (integrase), and PR (protease) [6]. An ENV-like (envelope) protein, which is typically identified in retroviruses, has also been found in a number of LTR retrotransposons [7,8]. LTR retrotransposons can be further classified into *Copia* and *Gypsy* superfamilies in plant genomes, according to the order in which the RT and IN appeared in the Pol region [5,9].

Because of the “copy and paste” mechanism for amplification of LTR retrotransposons, their copy number will be increased while active, they have been shown to make up the largest classes of TE content in the genome of most flowering plants, and they contribute greatly to the increase in genome size of their host genome [10]. For instance, the proportion of LTR retrotransposons was estimated to be 5.6% in the *Arabidopsis thaliana* genome (~125 Mb) [11], 22% of the *Oryza sativa* genome (~389 Mb) [12], and 75% of the *Zea mays* genome (~2.3 Gb) [13]. A previous study of the genome of a wild rice relative, *Oryza australiensis*, suggested that the burst and accumulation of three LTR retrotransposons families, namely *Kangourou, Wallabi*, and *RIRE1*, produced more than 90,000 copies within the past three million years (MY), increasing the size of the host genome two-fold. Proliferation of LTR retrotransposons in the maize genome also increased its genome size from 1.2 Gb to 2.4 Gb in nearly 3 MY [14]. As well as their impact on size variation in their host genome, LTR retrotransposons have also proven to play important roles in gene regulation [15,16], genome structural rearrangements [17], and other genetic functions. For instance, when an LTR element, *Gret1*, integrated close to the *VvmbyA1* genes in grapes, the grape skin color changed [15]. Another report suggested that insertion of *Rider* close to the *Ruby* gene introduced a novel regulatory element, which increased anthocyanin production, leading to the red fruit flesh of blood oranges [18].

*Morus* (mulberry) is the representative genus of the widespread plant family Moraceae (order Rosales). The *Morus* genus consists of 12–16 species, including more than 1000 cultivars, and *Morus* spp. are globally widespread [19,20].

The dynamics of the insertion and deletion processes of LTR retrotransposons in mulberry are poorly understood. The genome of *M. notabilis* C.K.Schneid is estimated to be ~357 Mb with 14 chromosomes (2*n* = 14), and represents the first completely sequenced mulberry genome, offering suitable reference genome sequences with which to analyze the evolutionary time-dynamics of LTR retrotransposons, including their insertion times, proliferation, and deletion [21]. In this regard, a genome-wide analysis of the evolutionary birth and death dynamic processes of LTR retrotransposons would significantly improve our understanding of the important roles played by TEs in mulberry genome evolution.

## 2. Results

### 2.1. Characterization of Long Terminal Repeat (LTR) Retrotransposons

**Figure 1:**
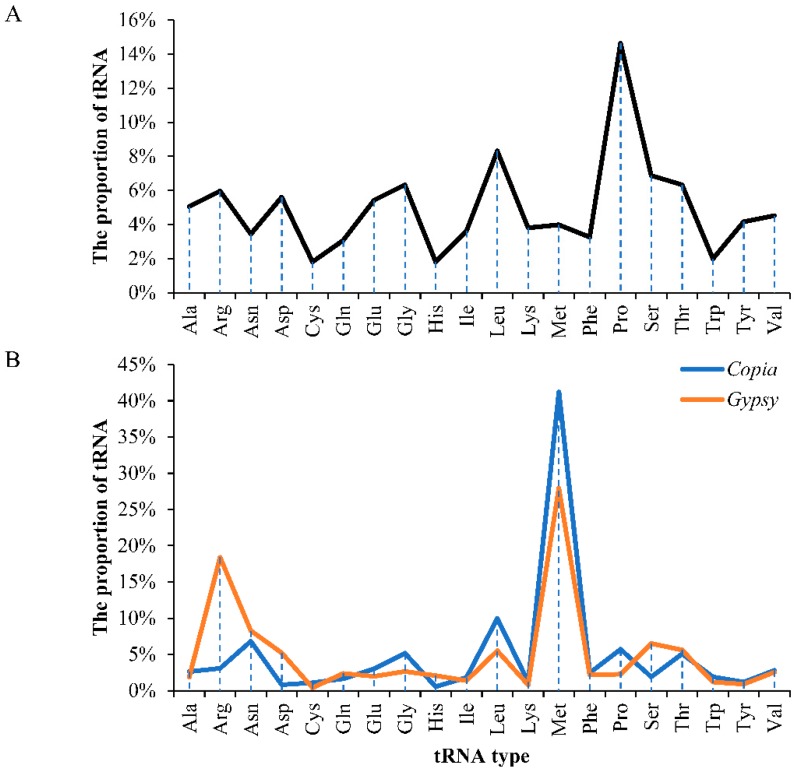
Statistics of tRNA usage in different superfamilies. (**A**) The proportion of different tRNAs in the mulberry genome; (**B**) Comparison of tRNA usage of different superfamilies. The *x*-axis denotes different tRNAs. The *y*-axis represents proportion of tRNA.

A total of 2916 full-length LTR retrotransposons were identified in the mulberry genome (Table 1). Among the 2916 elements, 1532 or 1384 elements were classified into the *Copia* or *Gypsy* superfamilies, respectively. These *Copia* and *Gypsy* elements were further classified into 202 and 114 families, respectively, according to the 80-80-80 rule reported previously [5]. The lengths of the full-length *Copia* elements were within the range from 1303 bp to 24,944 bp, and those of the LTRs were from 97 bp to 2853 or 2834 bp (Table 1 and Figure S1), while the lengths of the full-length *Gypsy* elements were from 1468 bp to 23,704 bp and those of the LTRs were within the range from 100 or 102 bp to 3352 or 3338 bp (Table 1 and Figure S1). Boundary feature analysis suggested that most of the *Copia* and *Gypsy* elements showed the canonical TG-CA boxes (Figure S2). The tRNA usage analysis results suggested that there was a significant tRNA usage preference through the PBS strings among the two superfamilies, *Copia* and *Gypsy* (Figure 1). As shown in Figure 1, we found that some tRNAs, including those carrying His, Lys, Cys, Trp, or Tyr, were seldom used as a primer of reverse transcription in both *Copia* and *Gypsy* elements. Most of the remaining tRNAs occurred at low frequencies. The tRNA*_Met_* was the most frequently used type in both superfamilies.

### 2.2. Phylogenetic Relationships

**Figure 2:**
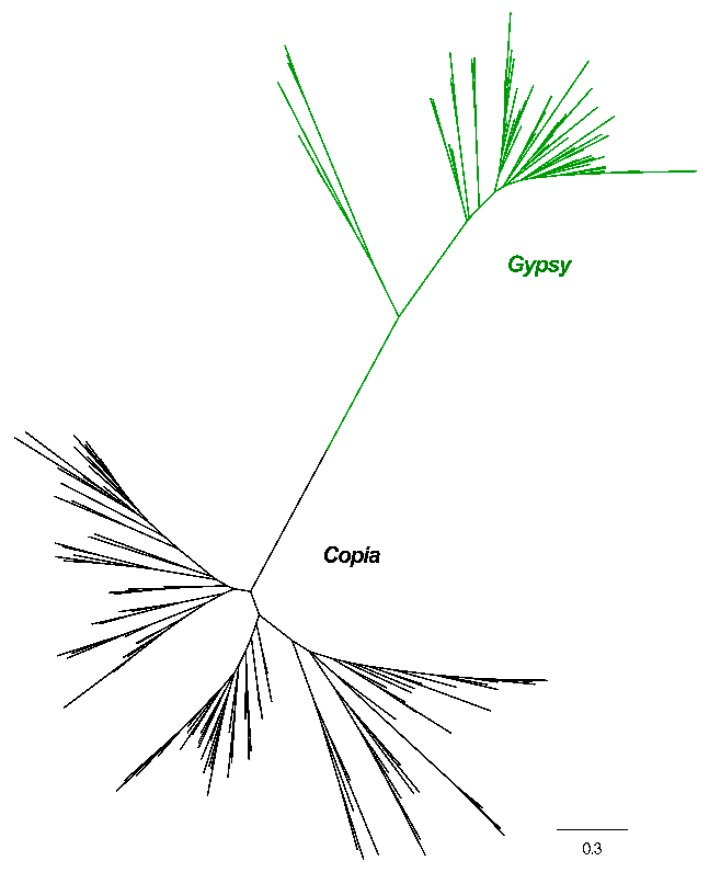
Phylogenetic relationships of *Copia* and *Gypsy* elements identified in the mulberry genome. Nucleotide sequences of reverse transcriptase (RT) of individual families were aligned by MUSCLE (v.3.8.31)**.** After best-fit models were evaluated by MEGA6, these sequences were used to construct the phylogenetic trees based on the maximum-likelihood method. Green branches, *Gypsy*; Black branches, *Copia*.

Phylogenetic trees of *Copia* and *Gypsy* elements were constructed based on their RT domain similarities for both types of elements. As shown in Figure 2 and Figure S3, the tree was clearly divided into two branches with perfect bootstrap values, which means that it was robust enough to classify LTR elements into the two different superfamilies based solely on the similarity of the RT sequences.

**Figure 3:**
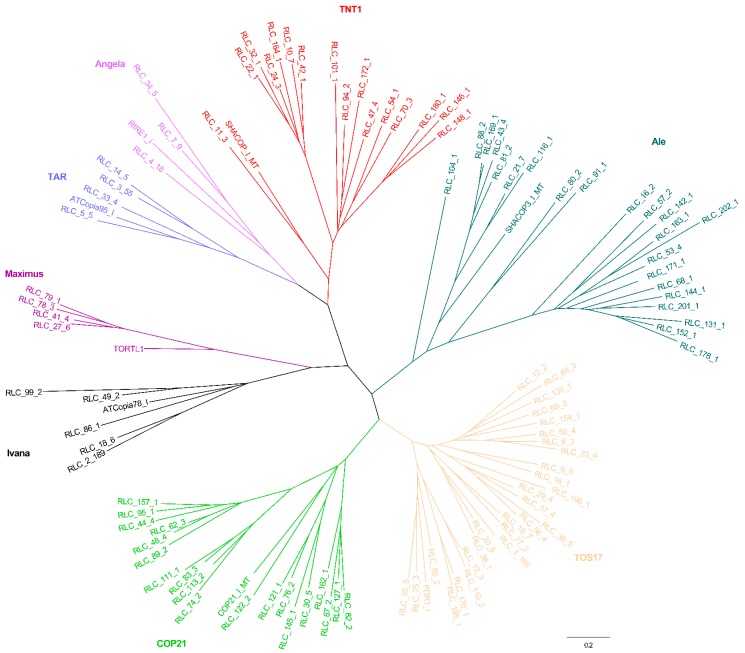
RT phylogenetic tree of *Copia* and representative members of the *Copia* lineages. Maximum-likelihood tree with representative RT sequences of each family and the representative members of *Copia* lineages. In the tree, each family is marked by its name. Representative sequences that were reported in previous studies were selected. Nucleotide sequences of RT of the individual families were aligned by MUSCLE (v.3.8.31). After best-fit models were evaluated by MEGA6, these sequences were used to construct the phylogenetic trees based on the maximum-likelihood method. The entire lineages are shown with different colors: Red, TNT1; Sapphire, TAR; Purple, Angela; Pale purple, Maximus; Black, Ivana; Green, COP21; Orange, TOS17; and Emerald green, Ale. Locus name of representative members of *Copia* lineages in Repbase (https://www.girinst.org/): ATCopia78_I, TORTL1, ATCopia95_I, RIRE1_I, SHACOP_I_MT, SHACOP3_I_MT, PDR1_I, and COP21_I_MT.

**Figure 4:**
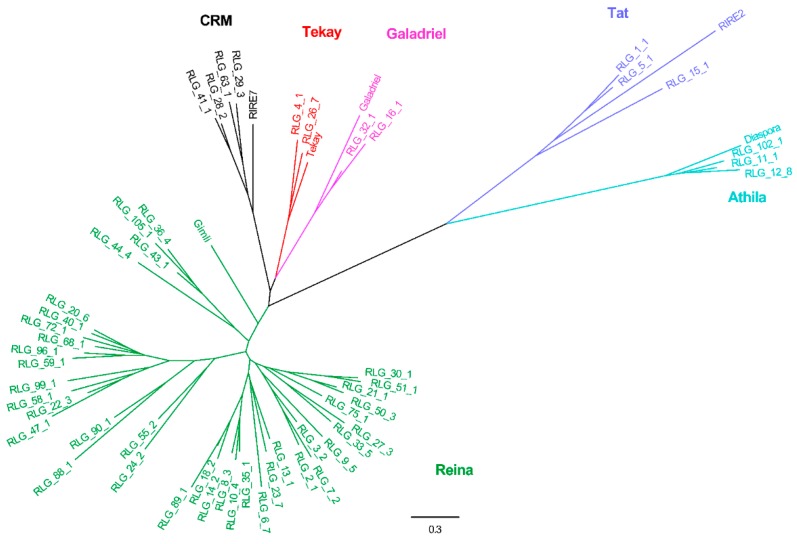
RT phylogenetic tree of *Gypsy* and representative members of the *Gypsy* lineages. Maximum-likelihood tree with representative RT sequences of each family and the representative members of the *Gypsy* lineages. In the tree, each family is marked by its name. Representative sequences that were reported in previous studies were selected. Nucleotide sequences of RT of individual families were aligned by MUSCLE (v.3.8.31). After best-fit models were evaluated by MEGA6, these sequences were used to construct the phylogenetic trees based on the maximum-likelihood method. The entire lineages are shown with different colors: Red, Tekay; Black, CRM; Green, Reina; Navy blue, Athila; Sapphire, Tat; and Purple, Galadriel. Locus name of representative members of the *Gypsy* lineages in Repbase (https://www.girinst.org/): Gimli, RIRE7, Galadriel, RIRE2, Diaspora, and Tekay.

Combined with other RT domains retrieved from known eukaryotic LTR lineage elements, an RT phylogenetic tree was constructed again. For the *Copia* phylogenetic tree, all elements were grouped into eight lineages, namely TAR, Maximus, Ivana, COP21, TOS17, Ale, TNT1, and Angela (Figure 3). For the *Gypsy* phylogenetic tree, all elements were classified into six lineages, namely CRM, Reina, Athila, Tat, Galadriel, and Tekay (Figure 4).

### 2.3. Insertion Time and Proportion Analysis

**Figure 5:**
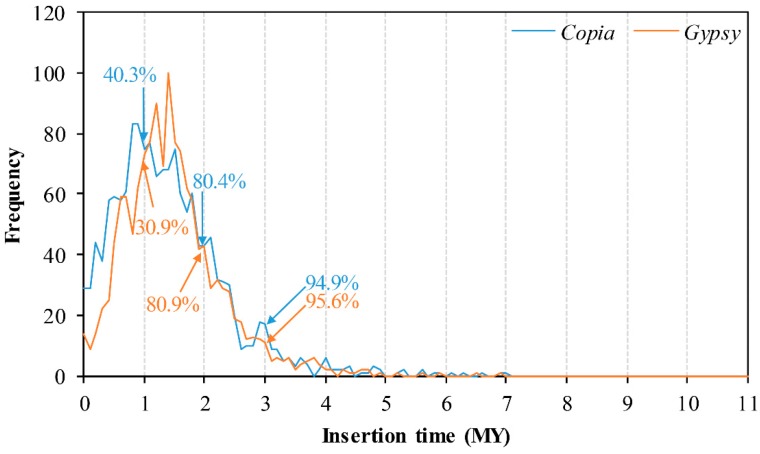
Overall insertion time distribution and amplification of full-length elements in the mulberry genome. Insertion times were split into bins of 0.1 MY. The *x*-axis denotes insertion time ranges for all full-length elements of *Copia* and *Gypsy*. The *y*-axis denotes the frequency of element insertions per time interval. The distribution pattern does not resemble an exponential distribution.

**Figure 6:**
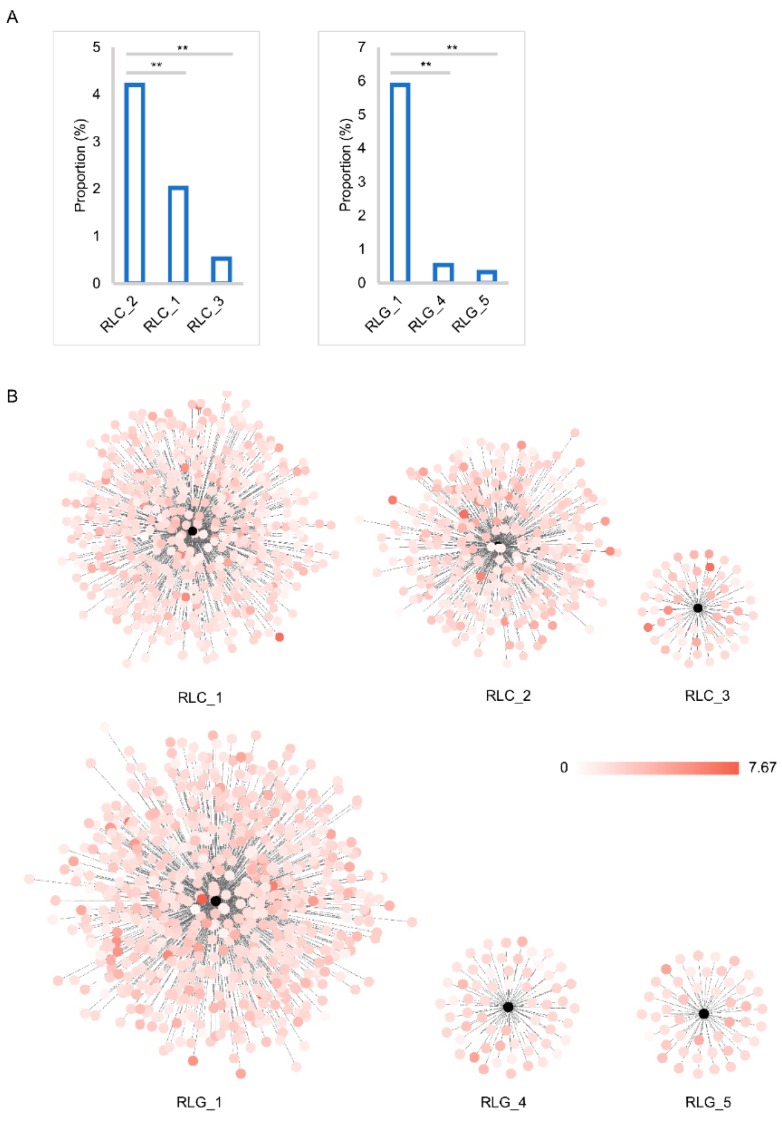
Insertion time and proportion analysis of each of three representative families of the *Copia* and *Gypsy* superfamilies. (**A**) proportional analysis of three representative families of the *Copia* (RLC_2, RLC_1, and RLC_3) and *Gypsy* (RLG_1, RLG_4, and RLG_5) superfamilies; The *x*-axis represents the families. The *y*-axis represents the proportion of the genome occupied by each family. (**B**) insertion time analysis of three representative families of the *Copia* (RLC_2, RLC_1, and RLC_3) and *Gypsy* (RLG_1, RLG_4, and RLG_5) superfamilies. Cytoscape (version 3.6.1) was used to construct the figure with yFiles layout model. Every black plot means a corresponding family. Other plots, whose colors range from white to red and are linked to the black plot, denote each member of the corresponding family. The insertion times of each member of one family were denoted by the colors of the plots. Color bar represents the insertion time range: 0 to 7.67 MY.

Insertion time analysis of all 2916 full-length elements indicated that nearly 81% of them had been inserted into the mulberry genome within the past 2 MY, while about 95% of them had appeared within the past 3 MY (Figure 5). Peak frequencies of *Copia* and *Gypsy* superfamily insertions were found at about 0.8 MY and 1.35 MY, respectively. A slight correlation relationship between the insertion time and the proportion of the genome occupied by members from both *Copia* and *Gypsy* superfamily was found (Figure S4). Then, the three highest proportion families of each of the *Copia* and *Gypsy* superfamilies were selected to analyze the relationship between its insertion time and the proportion of the genome occupied. Compared with other families from *Copia* and *Gypsy* superfamilies, RLC_2 and RLG_1 occupied the highest proportion of the mulberry genome, up to 4.20% and 5.88%, respectively (Figure 6A), followed by the RLC_1 (2.02%) and RLC_3 (0.53%) families from the *Copia* superfamily, while the proportion of the third-highest families was 0.54% and 0.32% for RLG_4 and RLG_5 in the *Gypsy* superfamily, respectively. Correlation analysis results suggested that there was a slight positive correlation between the insertion time of one element and the proportion of the genome that it occupied (Figure S4). Detailed insertion time analyses of the three highest proportion families of the *Copia* (908 members from the three most abundant families) and *Gypsy* superfamilies (783 members from the three most abundant families) suggested that these elements were inserted in the past 7.67 MY. The insertion time of different members from any one of the six families was estimated to cover a wide distribution range and could be grouped into several clusters (Figure 6B and Figure S5). The most abundant members from some families contributed mainly to the proliferation of these elements of the two superfamilies, *Copia* and *Gypsy* (Figures S4 and S6).

### 2.4. Selective Pressure Analysis

**Figure 7:**
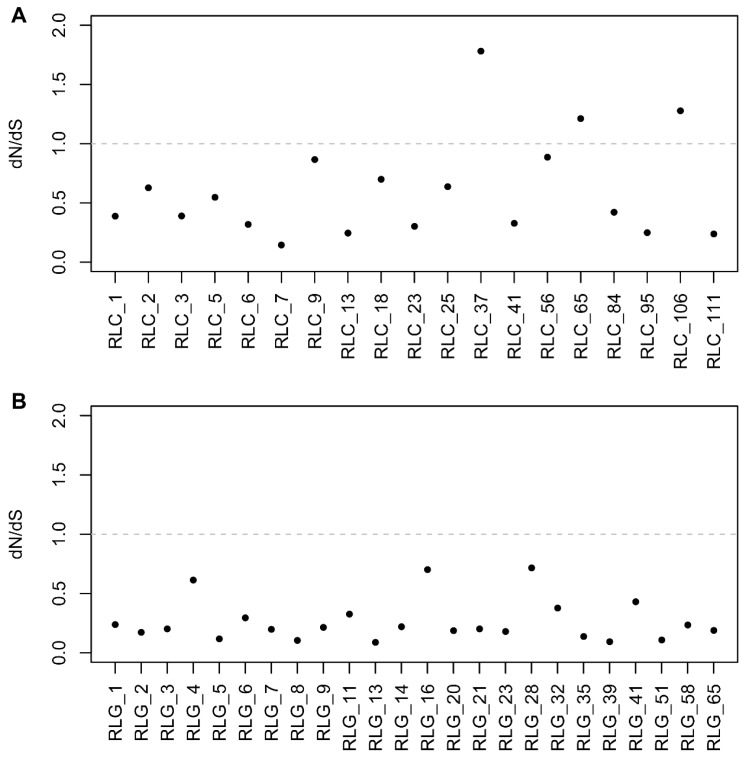
Nonsynonymous/Synonymous (dN/dS) rates for the LTR retrotransposons. (**A**) dN/dS rates for the *Copia* elements; (**B**) dN/dS rates for the *Gypsy* elements. The *x*-axis represents different families. The *y*-axis displays values of dN/dS. Only full-length sequences with intact RT genes were retained for selective pressure analysis. Protein sequences of RT genes were aligned by MUSCLE, then PAN2NAL was utilized to convert the protein MSA (multiple sequence alignment) format to a DNA codon-based alignment with the universal code model. The codeml module was used to perform dN/dS calculations. The rates of dN/dS reflected the selective pressures of these elements, dN/dS < 1, dN/dS = 1, and dN/dS > 1 denote purifying selection, neutral mutations, and adaptive molecular evolution, respectively.

The rates of nonsynonymous/synonymous (dN/dS) were used to estimate the selective pressure of these LTR elements. Nucleotide sequences of intact RT domains of full-length LTR retrotransposons were retrieved to analyze the selective pressure on these elements. A total of 19 families of the *Copia* superfamily were used to calculate the dN/dS rates, and the differences between dN/dS rates ranged from 0.1446 to 1.7807 (Figure 7A). Twenty-four families of the *Gypsy* superfamily were used to estimate the dN/dS rates, and the values of the dN/dS rates of the *Gypsy* superfamily ranged from 0.0887 to 0.7154 (Figure 7B). It is worth noting that the dN/dS rates of three families from the *Copia* superfamily were more than 1, namely 1.7807 (RLC_37), 1.2118 (RLC_65), and 1.2783 (RLC_106). Having a dN/dS >1 meant *Copia* elements from these three families were under positive selection pressure. On the other hand, the dN/dS rate values of all the *Gypsy* families were less than 1.

**Figure 8:**
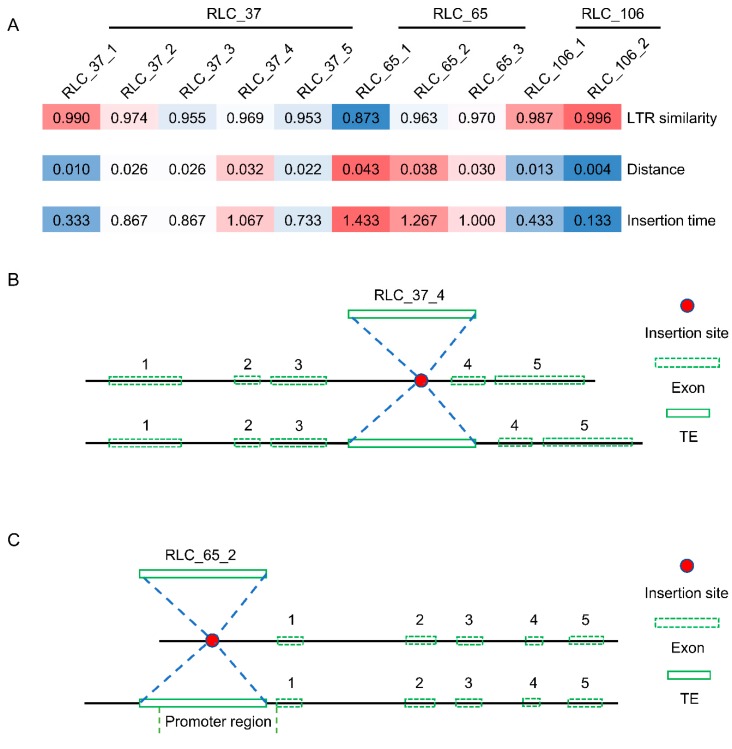
Insertion time and position analysis of three *Copia* families. (**A**) Insertion time analysis of the three *Copia* families. The “distance” here means the *K* values, which were used to calculate the insertion time using the equation *T* = *K*/*2r*; (**B**) Insertion position structure of the RLC_34_4 element. RLC_37_4 was inserted into the third intron of a mulberry gene (NCBI reference sequence, XM_010093293); (**C**) Insertion position structure of the RLC_65_2 element. RLC_65_2 was inserted into the promoter region of a mulberry gene (NCBI reference sequence, XM_010114426). Red plot means insertion site. Green box with dashed line means the exon. Numbers 1–5 correspond to exons 1–5. Green box with a solid line means the corresponding TE element. Figure 8B,C was drawn according to the actual length of each exon.

The three *Copia* families with dN/dS rates greater than 1 were selected for further analysis. The insertion time of all members of these three families ranged from 0.133 to 1.433 MY (Figure 8A). In other words, all these elements were young elements, and they shared a close similarity with respect to their LTRs (0.873 to 0.996). When it came to the position analysis of these elements in the mulberry genome, two members (RLC_37_4 and RLC_65_2) of the RLC_37 and RLC_65 families were used to illustrate the insertion position structure. As shown in Figure 8B and Figure S7, the RLC_37_4 inserted into the third intron of a mulberry gene and caused the longer intron of the gene. The other element, RLC_65_2, inserted into the promoter regions of one gene and introduced some *cis*-acting regulatory elements (Figure 8C and Figure S8 and Table S1). For example, circadian, a *cis*-acting regulatory element involved in circadian control, was found only in mulberry compared to three other close species (Figure S8).

## 3. Discussion

### 3.1. Evolutionary Landscape of Copia and Gypsy Elements

Nucleotide sequences of the RT-based phylogenetic analysis results suggested that the tree was clearly divided into two branches with perfect support (Figure 2). In other words, we can categorize *Copia* and *Gypsy* superfamilies to the level of superfamily based only on RT sequence similarity, a finding which is similar to previous reports [22,23].

A previous comparative analysis of *Copia* elements from Triticeae (20 families from wheat and barley), rice (46 families), and *Arabidopsis* (22 families) revealed six surprisingly conserved, ancient evolutionary lineages of *Copia* families before the divergence of dicots and monocots [24]. The six lineages were named as *Maximus*, *Ivana*, *Ale*, *Angela*, *TAR*, and *Bianca*, while the *Copia* elements were classified into ten clades in the *Medicago truncatula* genome [23]. Another comparative analysis of *Copia* elements from *Arabidopsis* (33 families), soybean (145 families), and rice (113 families) grouped these elements into seven lineages, namely *Maximus*, *Ivana*, *Ale*, *Angela*, *TAR*, *GMR*, and *Bianca* [22]. Six lineages of *Copia* elements from the banana (*Musa acuminata*) genome were classified, namely *Maximus*, *Angela*, *TONT1*, *TNT1*, *TOS17*, and *Hopscotch* [25]. Recently, comparative studies of *Copia* elements from eight AA-genome rice species also grouped these elements into six major lineages [26]. In the present work, further phylogenetic relationship analyses of *Copia* elements (202 families) suggested they can be grouped into eight lineages, namely *TAR*, *Maximus*, *Ivana*, *COP21*, *TOS17*, *Ale*, *TNT1*, and *Angela* (Figure 3). Combining the results from these previous studies with those from the present study, we considered that all previously characterized *Copia* families could be found in the mulberry genome.

When it came to phylogenetic analysis of the *Gypsy* elements in plant genomes, *Gypsy* lineages were mainly grouped into five or six lineages. For example, *Gypsy* elements were classified into six lineages in the *M. acuminate* genome (*Ogre* belongs to a lineage of plant LTR retrotransposons known as Tat [27]), namely *Tat*, *Athila*, *CRM*, *Reina*, *Tekay*, and *Galadriel* [25]. Phylogenetic studies of *Gypsy* elements classified these elements into five lineages (*Tat*, *Athila*, *CRM*, *Reina*, and *Tekay*), involving the *M. truncatula* (18 families), *Arabidopsis* (26 families), rice (125 families), and soybean (284 families) genomes [22,23]. The difference between the classification is the *Galadriel* lineage. Considering that *Galadriel* belongs to the chromoviridae branch [28], which is probably the most ancient phylogenetic pattern of *Gypsy* retroelements [29,30], our classification contained that lineage. As a result, 114 families of *Gypsy* elements in the mulberry genome could be grouped into six lineages.

### 3.2. Insertion and Deletion Dynamics of LTR Retrotransposons in the Mulberry Genome

We calculated the insertion times of all 2916 full-length elements, namely 1532 of *Copia* and 1384 *Gypsy* elements. About 95% of these elements inserted into the mulberry genome within the past 3 MY (Figure 5). This is mainly because of the “copy and paste” mechanism of retrotransposon amplification that, when new retrotransposons inserted and integrated into the host genome, some of these elements may be immediately amplified, increasing the copies of themselves after several rounds of bursting and accumulation [5]. In the active process of proliferation, the “copy and paste” mechanism of these elements in the host genome will be largely restricted by a number of mechanisms, such as unequal recombination, purifying selection, deletion, and methylation. These mechanisms are efficient ways of preventing TEs from inserting into gene coding regions and producing disadvantageous effects on gene function [1,11,14,31,32]. As a result, the distribution of these retrotransposons in their host genome was not random, being integrated into some distinct regions. Our results suggested that most of the *Copia* and all of the *Gypsy* elements were under strong purifying selection pressure, which meant that these elements experienced high levels of mutation and eventual deletion from the mulberry genome [33]. It is worth noting that three families of *Copia* elements were under positive selection (adaptive molecular evolution) pressure (Figure 7A), and insertion position structure analysis results indicated that these elements integrated mainly within the promoter or gene regions (Figure 8B,C, Figures S7 and S8), introducing some *cis*-acting regulatory elements to the promotors of genes (Table S1) or playing import roles in the evolution of some genes. As reported in previous studies, *Gypsy* elements tend to be clustered into the chromosomal centromeric regions [11,17,34], while other studies suggested that *Copia* elements were largely within and/or close to gene regions [35,36,37]. These results suggested that, of the two-retrotransposon superfamilies, the *Copia* elements may have the dominant influence on the evolution of some mulberry genes.

When we talk about the insertion of retrotransposons, it should be mentioned that although the insertion times and the proportion of the genome occupied by *Copia* and *Gypsy* elements showed a positive correlation, the insertion times of different members from one family was estimated to cover a wide range (Figure 6B and Figure S5). A possible interpretation for this phenomenon may be that some retrotransposons are activated and amplified as a newly burst branch under strong forces of natural selection, such as specific or unexpected environmental changes, including abiotic and biotic stresses [33]. Previous studies on some non-coding DNA elements (e.g., *mPing*, *dTstu1*, *mGing*, and *AhMITE1*) in plant genomes have shown that they can be activated under certain environmental conditions [38,39,40,41,42]. More efforts should be paid to reveal the precise activation mechanism of retrotransposons under different conditions in the future.

What is the deletion status of these retrotransposons? We attempted to reconstruct the insertion and deletion dynamics of these elements in the mulberry genome using previously reported methods in rice [24], Triticeae [24], *M. truncatula* [23], and soybean [22]. Assuming that these LTR retrotransposons are deleted from the genome at a constant deletion rate after they inserted into the genome, insertion time distribution of those full-length elements should be similar or exponential. So, the value of half-life rate can be used to evaluate the entire removal process in rice [24] and *M. truncatula* [23]. The overall age distribution of all full-length elements, either *Copia* or *Gypsy*, did not exhibit an exponential distribution at all (Figure 5). The distribution patterns were similar to those in Triticeae (wheat and barley), in which 86 *Copia* elements had been used to carry out the insertion time distribution analysis, and where the patterns also did not follow an exponential distribution [24]. In the present study, a total of 2916 full-length elements were used to analyze insertion time distribution patterns. Although an exponential distribution of insertion times was not reported, the large data set was enough to reflect a very long half-life of *Copia* and *Gypsy* elements in the mulberry genome.

## 4. Materials and Methods

### 4.1. Data Sources

The unmasked whole genomic sequence and gene annotation information of the mulberry genome were downloaded from the Morus Genome website (MorusDB, v 1.0, http://morus.swu.edu.cn/morusdb/) [43]. A mulberry tRNA database, which was used to predict the location of PBS (primer-binding site) of LTR elements, was also built by tRNAscan-SE (v.1.3.1) [44]. All full-length *Copia* and *Gypsy* elements were downloaded from MnTEdb [45]. The format of the family name was designated as RLC_#1_#2_Mno and RLG_#1_#2_Mno, where Mno denoted *Morus notabilis*, RL represented an LTR retrotransposon, C represented *Copia*, G denoted *Gypsy*, and #1 and #2 indicated the family number and the member number in the family, respectively [45]. RepeatMasker (v.4.0.3, http://www.repeatmasker.org) with RMBlast (Smith-Waterman cutoff, 255) was used to mine all relevant LTR sequences in the mulberry genome.

### 4.2. Sequence and Phylogenetic Analysis

Our own Perl script was used to retrieve 30 bp sequences upstream and downstream of all 5′LTRs of these elements according to the corresponding positions. Multiple sequence alignment was performed by MUSCLE (v.3.8.31) [46]. Weblogo (v.3, http://weblogo.threeplusone.com/) was used to generate the graphical representation of the multiple sequence alignment.

Nucleotide sequences of intact RT domains of full-length LTR retrotransposons were retrieved to analyze the selective pressure on these elements. Then, PAN2NAL was used to convert a multiple sequence alignment of proteins to a codon alignment of DNA sequences [47]. The codeml module, which was implemented in PAML, was utilized to perform selective pressure analyses [48].

Nucleotide sequences of isolated RT domains from intact LTR retrotransposons were aligned using MUSCLE (v.3.8.31) with default parameters [46]. The best-fit substitution models were estimated using MEGA6 [49]. According to these models, MEGA6 was used to construct the phylogenetic tree based on a maximum-likelihood method with bootstrap values set at 1000.

All Statistical analyses in this work were performed in R [50].

### 4.3. Estimation of Insertion Time

The two LTRs of intact LTR retrotransposons were identical when they inserted into the host genome [14]. According to previous research by Ma et al., the insertion times of intact LTR retrotransposons elements could be calculated by comparing their nucleotide divergence of the two LTRs [12]. Two LTRs of each full-length LTR retrotransposon were retrieved by our own Perl script and aligned using MUSCLE (v.3.8.31) [46]. Then, the baseml module, which was implemented in PAML [48], was utilized to estimated nucleotide divergence between the two LTRs. The insertion time (*T*) was calculated by the equation *T* = *K*/*2r*, where *r* = 1.3 × 10^−8^ per site per year [51], and *K* represented the divergence of the LTRs from the intact LTR retrotransposons.

## 5. Conclusions

The evolutionary dynamics of *Copia* and *Gypsy* elements in the mulberry genome are largely unknown. Here, we performed a comprehensive investigation and analysis of LTR retrotransposons in the mulberry genome, including their classification, insertion times, and evolutionary dynamics. All 2916 full-length elements were classified into 202 families of *Copia* and 114 families of *Gypsy.* About 95% of the copies had been integrated into the mulberry genome within past 3 MY. This present study provides new insights into the insertion and deletion dynamics of LTR retrotransposons in the mulberry genome. *Copia* and *Gypsy* elements exhibited a very long half-life in the mulberry genome. Further studies will be focused on the activation mechanisms of retrotransposons and the important roles TEs play in the architecture of the mulberry genome.

## Figures and Tables

**Table 1:** Summary of the *Copia* and *Gypsy* long terminal repeat (LTR) families.

Type	*Copia*	*Gypsy*
Full-length number	1532	1384
Family number	202	114
Full length (bp) ^a^	7829 (1303–24,944)	9526 (1468–23,704)
GC content	0.41	0.41
5′LTR length (bp) ^a^	404 (97–2853)	653 (102–3338)
3′LTR length (bp) ^a^	404 (97–2834)	653 (100–3352)

^a^ denotes mean (min–max).

## Supplementary Materials

The following are available online at https://www.mdpi.com/2073-4425/10/4/285/s1, Supplementary Table S1. Cis-acting regulatory element contained in the RLC_65_2 element. All cis-acting regulatory elements were predicted by PlantCARE website (http://bioinformatics.psb.ugent.be/webtools/plantcare/html/). Supplementary Figure S1. Correlation between size of LTR and LTR retrotransposons in different superfamilies. x-axis denotes length of 5′LTR; y-axis denotes length of entry element. Supplementary Figure S2. Sequence logo LTR border of LTR retrotransposons. A, Copia elements; B, Gypsy elements. The sequences were aligned using MUSCLE (version 3.8.31) under default parameters. Sequence logos of these sequences were produced by WebLogo (v.3). Supplementary Figure S3. Phylogenetic relationships of Copia and Gypsy elements identified in mulberry genome. Nucleotide sequences of RT of individual families were aligned by MUSCLE (v3.8.31). After best-fit models were evaluated by MEGA6, these sequences were used to construct the phylogenetic trees based on the maximum-likehood method. Green branches, Gypsy. Black branches, Copia. The display range of bootstrap values was set as 0 to 1. Supplementary Figure S4. Correlation of insertion time and proportion in the genome of full-length elements of the Copia and Gypsy superfamilies. x-axis denotes insertion times of these elements; y-axis represents the proportion of the genome. MY, million years. Supplementary Figure S5. Insertion time cluster analysis of the two largest families of each of the Copia and Gypsy superfamilies. Supplementary Figure S6. Insertion time of the highest proportion families of the Copia and Gypsy superfamilies. Supplementary Figure S7. Gene structure comparison between four closely related species. The RLC_37_4 was inserted into the third intron of mulberry gene XM_010093293 (NCBI reference sequence, XM_010093293). The homologous genes from three other close mulberry species, namely Prunus persica, Malus x domestica, and Pyrus x bretschneideri, were retrieved from The GDR database (https://www.rosaceae.org/) with accession numbers ppa009046m, MD13G1060300 and rna10415-v1.1-pbr, respectively. The figure was constructed by GSDS (http://gsds.cbi.pku.edu.cn/index.php, Gene Structure Display Server). Supplementary Figure S8. Cis-acting regulatory elements analysis. All cis-acting regulatory elements were predicted by PlantCARE website (http://bioinformatics.psb.ugent.be/webtools/plantcare/html/). The RLC_65_2 was inserted into the promoter region of a mulberry gene (NCBI reference sequence, XM_010114426). The promoter sequences of homologous genes from three other close mulberry species, namely Prunus persica, Malus x domestica, and Pyrus x bretschneideri, were retrieved from the GDR database (https://www.rosaceae.org/) with accession numbers ppa002853m, MD11G1298100 and rna51474-v1.1-pbr, respectively. y-axis means types of motifs. x-axis denotes numbers of motifs in the promoter region.

## Abbreviations

LTR	Long terminal repeat
TE	transposable element
PBS	primer-binding site
PPT	polypurine tract
RT	reverse transcriptase
RH	RNase H
IN	integrase
PR	protease
MY	million years
